# Practical Synthesis of Hydroxychromenes and Evaluation of Their Biological Activity

**DOI:** 10.3390/molecules17010240

**Published:** 2011-12-28

**Authors:** Jae-Chul Jung, Seikwan Oh

**Affiliations:** 1 Institute of Life Science Research, Rexgene Biotech, Ochang, Chungbuk 363-838, Korea; 2 Department of Neuroscience and TIDRC, School of Medicine, Ewha Womans University, Seoul 158-710, Korea

**Keywords:** anti-inflammatory, brodifacoum, coupling reaction, difethialone, Friedel-Crafts acylation, intramolecular ring cyclization reaction

## Abstract

A simple and efficient seven steps synthesis of brodifacoum and difethialone from phenylacetyl chloride is described. The key synthetic strategies involve Friedel-Crafts acylation, intramolecular ring cyclization and condensation reaction in the presence of Brønsted-Lowry acids. It was found that brodifacoum showed favorable inhibiting activities on LPS-stimulated nitrite production in BV-2 microglia cells. Brodifacoum exhibited superior anti-inflammatory effects than difethialone. We expect that an efficient linear synthesis of 4-hydroxycoumarin derivatives and key fragments that are useful agents for the modulation of inflammation as well as inhibition of coagulation will be of practical use.

## 1. Introduction

4-Hydroxycoumarin-based anticoagulants such as the first generation anticoagulant warfarin and its related second generation anticoagulants such as brodifacoum, bromadiolone, chlorophacinone, difenacoum, coumatetralyl, flocoumafen, and difethialone are effective anticoagulant rodenticides. The anticoagulant rodenticides difenacoum and brodifacoum showed a plasma elimination half-life of 91.7 days. In general, the elimination half-lives in plasma for first-generation rodenticides were shorter than those for second-generation rodenticides. These results revealed that the superwarfarins such as difenacoum, brodifacoum, flocoumafen, and difethialone showed higher anticoagulant effect than that of warfarin [1].

**Figure 1:**
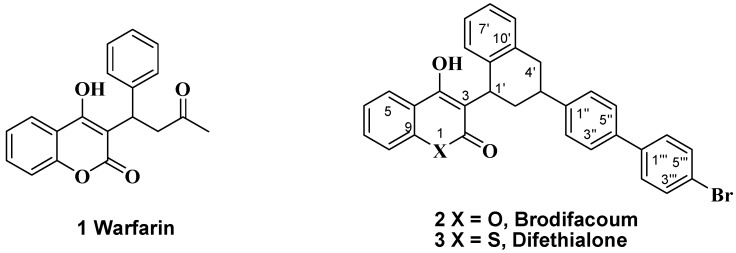
Structures of warfarin (**1**), brodifacoum (**2**), and difethialone (**3**).

Recently, a number of comparative pharmacological investigations of 4-hydroxycoumarin derivatives have shown good anticoagulant activity with low side effects and little toxicity (Figure 1). In addition, they have evoked a great deal of interest due to their biological properties and characteristic conjugated molecular architecture. Many of them possess important pharmacological efficacies including analgesic [2], anti-arthritis [3], anti-inflammatory [4], anti-pyretic [5], anti-bacterial [6], anti-viral [7], and anti-cancer [8] effects.

4-Hydroxycoumarin and its derivatives have been effectively used as anticoagulants for the treatment of disorders in which there is excessive or undesirable clotting, such as thrombophlebitis [9], pulmonary embolisms [10], and certain cardiac conditions [11]. The Dimitra group [12] reported that several coumarin derivatives showed inhibition of lipid peroxidation and scavenge of reactive oxygen radicals (ROS) *in vivo* related with potent inhibition of cyclooxygenase-1 (COX-1). The Stasi group [13] described how 4-hydroxycoumarin and its derivatives showed good anti-inflammatory activity, including inhibition of lipid peroxidation and scavenge of superoxide radicals in intestinal track.

**Scheme 1:**
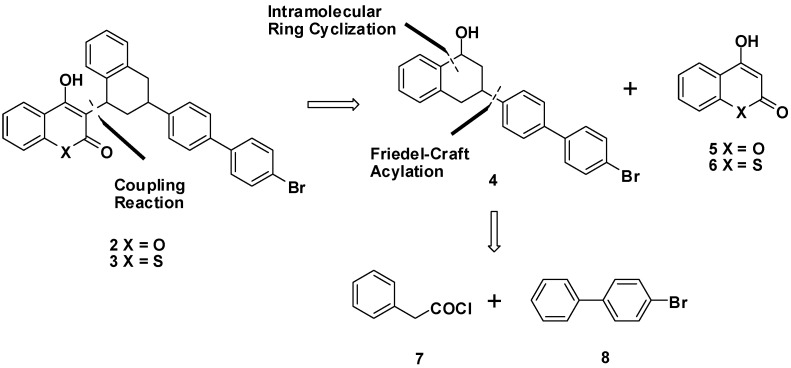
Retrosynthetic analysis of the 4-hydroxycoumarins.

The skeletons of 2*H*-1-benzopyran-2-ones **5–6** and 3-(*p*-bromobiphenyl-4-yl)-1,2,3,4-tetrahydronaphthalen-1-ol (**4**) are essential structural features for brodifacoum (**2**) and difethialone (**3**) (Scheme 1). In general, 4-hydroxycoumarin derivatives have traditionally been coupled using Brønsted-Lowry acids such as hydrochloric acid or sulfuric acid. Our synthetic approach was focused to construction of C3-C1′ and C9′-C1′ formation through a condensation reaction in the presence of AcOH/H_2_SO_4_ and intramolecular ring cyclization reaction. In addition, to establish C3′-C1′′ bond formation the Friedel-Crafts acylation reaction performed in good yield using phenylacetyl chloride (**7**) with 4-bromobiphenyl (**8**) in the presence of aluminum chloride. In a continuation of our medicinal research program dealing with the synthesis of biologically active 4-hydroxycoumarin derivatives, we would like to report a simple synthesis of brodifacoum {3-[3-[4-(4-bromophenyl)phenyl]-1,2,3,4-tetrahydronaphthalen-1-yl]-2-hydroxychromen-4-one} and difethialone {3-[3-[4-(4-bromophenyl)-phenyl]-1-tetralinyl]-2-hydroxy-4-thiochromenone} through acylation, intramolecular ring cyclization, and coupling reactions. They were then evaluated for suppression of LPS-induced NO generation as markers of anti-inflammation in cultured cells. The results suggest that they could be possible lead molecules for anti-inflammatory agents.

## 2. Results and Discussion

### 2.1. Chemistry

**Scheme 2:**
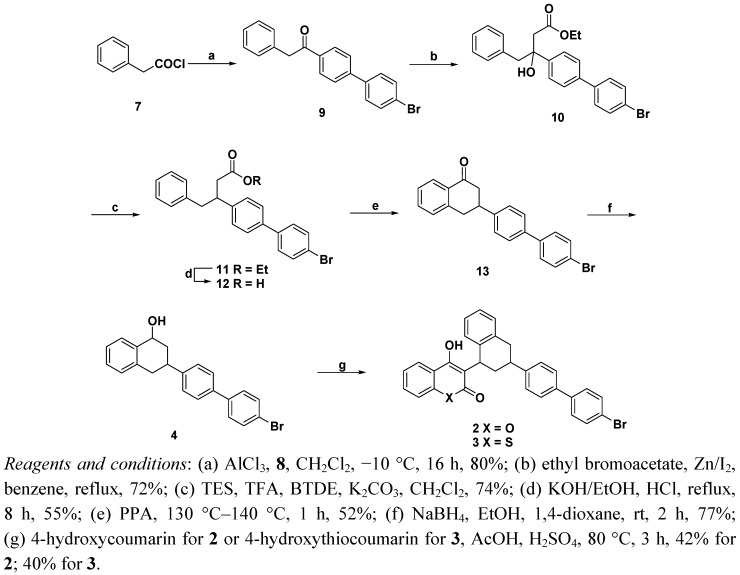
Synthesis of brodifacoum (**2**) and difethialone (**3**).

To generate the key intermediate 3-(*p*-bromobiphenyl-4-yl)-1,2,3,4-tetrahydronaphthalen-1-one (**13**) from 1-(*p*-bromobiphenyl-4-yl)-2-phenylethanone (**9**) the ketone **7** was prepared from 2-phenylacetyl chloride and 4-bromobiphenyl (**8**) under Friedel-Crafts conditions [14] (Scheme 2).

Compound **7** underwent a Reformatsky reaction with ethyl bromoacetate in the presence of zinc and iodine to give the tertiary alcohol **10** [16], which was dehydroxylated by using triethylsilane (TES), trifluoroacetic acid (TFA), and boron trifluoride diethyl etherate (BTDE) in dichloromethane to afford ester **11**, which was then subsequently treated with potassium hydroxide in ethanol to give acid **12** in 41% (two steps) yield. Interestingly, acid hydrolysis of ester **11** using 6 N HCl with acetic acid in ethanol afforded to acid **12** with low yield due to the generation of decompositon products.

Acid **12** underwent intramolecular ring cyclization using polyphosphoric acid (PPA) at 130 °C–140 °C to generate tetralone **13** in 52% yield. Reduction was accomplished with sodium borohydride (NaBH_4_) in ethanol to give secondary alcohol **4** in 77% yield. Finally, the secondary alcohol **4** was coupled with 4-hydroxycoumarin or 4-hydroxythiocoumarin in the presence of acetic acid and sulfuric acid at 80 °C to yield brodifacoum and difethialone in 42% and 40% yields, respectively.

### 2.2. Biology

**Figure 2:**
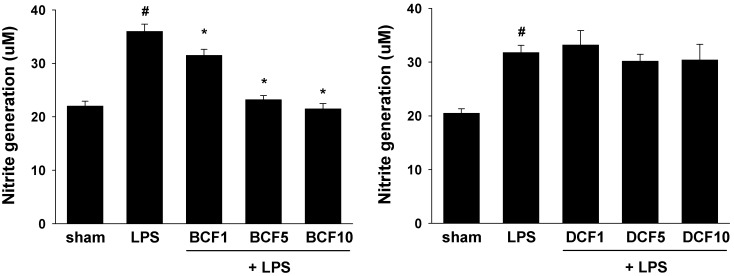
Suppression of NO production in LPS-treated microglia. The cells were treated with 1 µg/mL of LPS only or LPS plus different concentrations (1, 5, and 10 µg/mL) of compounds at 37 °C for 24 h. At the end of incubation, 50 µL of the medium was removed to measure nitrite production. All values represent mean ± S.E. of three independent experiments performed in triplicate. ^#^* p* < 0.05 indicates significant difference compare with sham group. * *p* < 0.05 indicates significant difference compare with LPS group.

Nitrite was used as a measure of NO production. The suppression of LPS-induced NO generation by the prepared 4-hydroxycoumarin derivatives in cultured cells was evaluated by the published test method [15] and the results are summarized in Figure 2. Brodifacoum (**2**, BCF) one of the 4-hydroxycoumarin derivatives, inhibited nitrite accumulation in LPS-stimulated microglia BV-2 cells, while difethialone (**3**, DCF) showed no activity on inhibition of NO production at 1–10 µg/mL concentrations in LPS-stimulated BV-2 cells (Figure 2). These results also showed that brodifacoum possessed good anti-inflammatory activity. Neither brodifacoum nor difethialone alone showed cytotoxicity or affect NO generation in BV2 cells with the concentration of 10 µg /mL [16].

## 3. Experimental

### 3.1. General

Reactions requiring anhydrous conditions were performed with the usual precautions for rigorous exclusion of air and moisture. Tetrahydrofuran was distilled from sodium benzophenone ketyl prior to use. Thin layer chromatography (TLC) was performed on precoated silica gel G and GP uniplates from Analtech and visualized with 254-nm UV light. Flash chromatography was carried out on silica gel 60 [Scientific Adsorbents Incorporated (SAI), particle size 32–63 µm, pore size 60 Å]. ^1^H-NMR, and ^13^C-NMR spectra were recorded on Bruker DPX 400/500 instruments at 400/500 MHz and 100/125 MHz; respectively. The chemical shifts are reported in parts per million (ppm) downfield from tetramethylsilane, and *J*-values are in Hz. Infrared (IR) spectra were obtained on an ATI Mattson FT/IR spectrometer. Mass spectra were recorded with a Waters Micromass ZQ LC-Mass system and high resolution mass spectra (HRMS) were measured with a Bruker BioApex FTMS system by direct injection using an electrospray interface (ESI). When necessary, chemicals were purified according to the reported procedures [17].

*1-(p-Bromobiphenyl-4-yl)-2-phenylethanone* (**9**). To a stirred solution of phenylacetyl chloride (**7**, 1.0 g, 6.5 mmol) and 4-bromobiphenyl (**8**, 1.0 g, 4.3 mmol) in dichloromethane (16 mL) was added portionwise aluminum chloride (0.8 g, 6.0 mmol) at −10 °C and then the mixture was stirred at same temperature for 16 h. The reaction mixture was poured to aqueous 3 N HCl solution (8 mL) and extracted with dichloromethane (10 mL × 3). The combined organic layer was washed with saturated aqueous NH_4_Cl solution (15 mL) and organic phase was separated, dried over anhydrous MgSO_4_, filtered, and concentrated under reduced pressure. The residue was purified by flash column chromatography (silica gel, ethyl acetate/*n*-hexanes = 1:2, v/v) to afford **9** (1.83 g, 80%) as a white solid. mp 93.1 °C. IR (neat, NaCl) 1679, 1601, 1482, 1222, 1076, 808 cm^–1^; ^1^H-NMR (500 MHz, CDCl_3_) d 8.08 (d, 2H, *J* = 6.5 Hz, aromatic-H), 7.65–7.57 (m, 4H), 7.49–7.44 (m, 2H), 7.36–7.22 (m, 5H), 4.31 (s, 2H); ^13^C-NMR (125 MHz, CDCl_3_) d 197.1, 144.5, 139.2, 138.7, 135.5, 134.5, 132.2, 132.0, 129.5, 129.3, 128.8, 128.7, 128.6, 128.5, 127.6, 127.1, 126.9, 126.8, 126.7, 45.6; HRMS calcd. for C_2__0_H_15_BrNaO: 373.0204 [M+Na]^+^, found: 373.0137.

*Ethyl 3-(p-bromobiphenyl-4-yl)-4-phenylbutanoate* (**11**). To a stirred solution of tertiary alcohol (**10**, 1.1 g, 2.5 mmol) in dichloromethane (6.0 mL) was added triethylsilane (0.6 g, 5.2 mmol), boron trifluoride diethyl etherate (4 µL) and trifluoroacetic acid (2.1 g, 18.3 mmol) at room temperature. The reaction mixture was refluxed for 8 h and washed with 30% aqueous potassium carbonate solution (8.5 mL). The organic phase was separated and washed with sat’d sodium bicarbonate solution (10 mL) and brine (10 mL). The organic layer was separated and dried over anhydrous MgSO_4_, filtered, and concentrated under reduced pressure to give ester **11** (0.78 g, 74%) as a white solid. mp 62 °C. IR (neat, NaCl) 1732, 1481, 1149, 1001, 805 cm^–1^; ^1^H-NMR (500 MHz, CDCl_3_) d 7.58–7.38 (m, 7H), 7.26–7.17 (m, 4H), 7.09 (d, 2H, *J* = 7.4 Hz, aromatic-H), 4.03 (d, 2H, *J* = 7.5 Hz, CH2), 3.51–3.43 (m, 1H), 2.92–2.99 (m, 2H), 2.73–2.60 (m, 2H), 1.23 (t, 3H, *J* = 7.5 Hz, CH3); ^13^C-NMR (125 MHz, CDCl_3_) d 172.2, 143.1, 139.8, 139.4, 138.1, 131.9, 131.8, 129.3, 129.1, 128.8, 128.6 (2C), 128.5, 128.3, 128.1, 126.8, 126.2, 126.0, 121.4, 60.4, 43.6, 43.0, 40.3, 14.1; HRMS calcd. for C_24_H_2__4_BrO_2_: 423.0960 [M+H]^+^, found: 423.2313.

*3-(*p*-Bromobiphenyl-4-yl)-1,2,3,4-tetrahydronaphthalen-1-one* (**13**). To a stirred solution of acid (**12**, 2.5 g, 6.2 mmol) was added polyphosphoric acid (6.0 g) in MeOH (4.0 mL) and the mixture was stirred at 130 °C–140 °C for 1 h. The reaction mixture was cooled at room temperature and extracted with dichloromethane (10 mL). The organic layer was washed with 10% aqueous sodium bicarbonate solution (8.5 mL) and water (10 mL). The organic phase was separated, dried over anhydrous MgSO_4_, filtered, and concentrated under reduced pressure to give ketone **13** (1.2 g, 52%) as a white solid. mp 158 °C. IR (neat, NaCl) 3031, 2922, 1698, 1611, 1584, 1512, 1454, 1244, 1066, 824 cm^–1^; ^1^H-NMR (CDCl_3_, 400.13 MHz) d 7. 70 (d, 1H, *J* = 7.5 Hz), 7.62–7.44 (m, 7H), 7.38–7.21 (m, 4H), 3.69–3.75 (m, 1H), 3.02–3.18 (m, 4H); HRMS calcd. for C_2__2_H_18_BrO: 377.0541 [M+H]^+^, found: 377.1124.

*3-(*p*-Bromobiphenyl-4-yl)-1,2,3,4-tetrahydronaphthalen-1-ol* (**4**). To a stirred solution of ketone (**13**, 1.0 g, 2.6 mmol) in ethanol (4.0 mL) and 1,4-dioxane (4.0 mL) was added sodium borohydride (0.14 g, 3.7 mmol) and the mixture was stirred at room temperature for 2 h. The reaction mixture was quenched with water (5 mL) and acidified with aqueous 20%-HCl solution and then extracted with dichloromethane (7 mL × 3). The combined organic phases were washed with sat’d sodium bicarbonate solution (15 mL) and brine (15 mL). The organic layer was separated, dried over MgSO_4_, and concentrated under reduced pressure to give secondary alcohol, which was purified by flash column chromatography to give compound **4** (0.76 g, 77%) as a white solid. mp 151 °C. IR (neat, NaCl) 3363, 1482, 1389, 1000, 826 cm^–1^. ^1^H-NMR (CDCl_3_, 400.13 MHz) d 7. 64 (d, 1H, *J* = 12.5 Hz), 7.60–7.55 (m, 4H), 7.49–7.44 (m, 2H), 7.40–7.34 (m, 2H), 7.32–7.21 (m, 2H), 7. 13 (d, 1H, *J* = 12.8 Hz), 5.10–5.49 (m, 1H), 3.22–2.88 (m, 3H), 2.61–2.59 (m, 1H), 1. 85 (d, 1H, *J* = 8.5 Hz); HRMS calcd. for C_2__2_H_19_BrNaO: 401.0517 [M+Na]^+^, found: 401.1025.

*Brodifacoum* (**2**) a *Difethialone* (**3**). To a stirred solution of alcohol (**4**, 4.6 g, 12.1 mmol) and 98% sulfuric acid (0.3 mg) in acetic acid (7.5 mL) was added 4-hydroxycoumarin or 4-hydroxythiocoumarin (12.7 mmol) at room temperature and then the reaction mixture was stirred at 80 °C for 3 h. The solvent was removed under reduced pressure and the residue was treated with dichloromethane (10 mL) and water (10 mL). The organic phase was separated, dried over anhydrous MgSO_4_, filtered, and concentrated under reduced pressure. The residue was purified by flash column chromatography (silica gel, ethyl acetate/*n*-hexanes = 1:2, v/v) to give brodifacoum (**2**, 2.7 g, 42%) and difethialone (**3**, 2.6 g, 40%), respectively. Brodifacoum (**2**): White solid. mp 227 °C. IR (neat, NaCl) *v* 3355 (OH), 2986, 1665 (C=O), 1608 (C=C), 1525, 1420, 1242 1045, 823 cm^–1^. ^1^H-NMR (CDCl_3_, 400.13 MHz) d 7. 62 (dd, 2H, *J* = 8.5, 8.5 Hz), 7.68–7.33 (m, 12H), 7.21–6.83 (m, 2H), 4.90–4.83 (m, 1H), 3.28–3.02 (m, 1H), 2.91–2.80 (m, 1H), 2.70–2.49 (m, 2H), 2.31–2.10 (m, 1H); HRMS calcd. for C_31_H_23_BrNaO_3_: 545.0728 [M+Na]^+^, found: 545.0825. Difethialone (**3**): Beige solid. mp 228 °C. IR (neat, NaCl) 3422 (OH), 1668 (C=O), 1610, 1510, 1433, 1261, 1068, 835 cm^–1^. ^1^H-NMR (CDCl_3_, 400.13 MHz) d 7. 58 (dd, 2H, *J* = 8.5, 8.0 Hz), 7.46–6.68 (m, 14H), 4.85–4.76 (m, 1H), 3.19–3.04 (m, 1H), 2.84–2.69 (m, 1H), 2.67–2.51 (m, 1H), 2.46–2.38 (m, 1H), 2.16–2.01 (m, 1H); HRMS calcd. for C_31_H_23_BrNaO_2_S: 561.0500 [M+Na]^+^, found: 561.0422.

### 3.2. BV-2 Microglia Culture

The murine BV-2 microglia cell line was maintained in DMEM supplemented with 10% FBS and penicillin/streptomycin at 37 °C in a humidified incubator under 5% CO_2_. For all experiments, cells were plated at a density of 1 × 10^5^ cells/mL in 24-well plates.

### 3.3. Nitric Oxide Assay

When the cells were activated by the exposure of LPS, generated NO was accumulated in the form of nitrite in the cultured medium. The Griess reaction was used to perform nitrite assays. Cells were incubated with LPS (lipopolysaccharide, 100 ng/mL) and various concentrations of modafinil derivatives for 24 h at 37 °C. The culture media was then mixed with an equal volume of reagent (1 part 0.1% *N*-1-naphthylethylenediamine dihydrochloride, 1 part 1% sulfanilamide in 5% phosphoric acid) in 96-well plates. The absorbance was determined at 540 nm as level of nitrite accumulation using a microplate reader. Data are reported as the mean ± the standard deviation of three observations.

## 4. Conclusions

In conclusion, we have demonstrated a simple and practical synthetic route to brodifacoum and difethialone using readily available inexpensive reagents and simple reaction conditions that do not require any special equipment or techniques. We expect that this method would be useful for the practical preparation of brodifacoum and difethialone and modification of its derivatives. We determined their biological activities in cultured cells and found that brodifacoum showed favorable inhibitory activities on LPS-stimulated nitrite production in BV-2 microglia cells, and that the anti-inflammatory activity of brodifacoum was better than that of difethialone. These results suggest that brodifacoum could be a useful lead molecule for the development of anti-inflammatory agents.
